# Quantifying the Adaptive Potential of an Antibiotic Resistance Enzyme

**DOI:** 10.1371/journal.pgen.1002783

**Published:** 2012-06-28

**Authors:** Martijn F. Schenk, Ivan G. Szendro, Joachim Krug, J. Arjan G. M. de Visser

**Affiliations:** 1 Institute for Genetics, University of Cologne, Köln, Germany; 2 Laboratory of Genetics, Wageningen University, Wageningen, The Netherlands; 3 Institute for Theoretical Physics, University of Cologne, Köln, Germany; 4 Systems Biology of Ageing Cologne (Sybacol), University of Cologne, Köln, Germany; Uppsala University, Sweden

## Abstract

For a quantitative understanding of the process of adaptation, we need to understand its “raw material,” that is, the frequency and fitness effects of beneficial mutations. At present, most empirical evidence suggests an exponential distribution of fitness effects of beneficial mutations, as predicted for Gumbel-domain distributions by extreme value theory. Here, we study the distribution of mutation effects on cefotaxime (Ctx) resistance and fitness of 48 unique beneficial mutations in the bacterial enzyme TEM-1 β-lactamase, which were obtained by screening the products of random mutagenesis for increased Ctx resistance. Our contributions are threefold. First, based on the frequency of unique mutations among more than 300 sequenced isolates and correcting for mutation bias, we conservatively estimate that the total number of first-step mutations that increase Ctx resistance in this enzyme is 87 [95% CI 75–189], or 3.4% of all 2,583 possible base-pair substitutions. Of the 48 mutations, 10 are synonymous and the majority of the 38 non-synonymous mutations occur in the pocket surrounding the catalytic site. Second, we estimate the effects of the mutations on Ctx resistance by determining survival at various Ctx concentrations, and we derive their fitness effects by modeling reproduction and survival as a branching process. Third, we find that the distribution of both measures follows a Fréchet-type distribution characterized by a broad tail of a few exceptionally fit mutants. Such distributions have fundamental evolutionary implications, including an increased predictability of evolution, and may provide a partial explanation for recent observations of striking parallel evolution of antibiotic resistance.

## Author Summary

Although mutations with positive effect on fitness are rare, they contribute disproportionally to evolution, because they are favored by natural selection. Because they are rare and hard to study experimentally, little is known about the frequency and variation in fitness effects of beneficial mutations, and it is often assumed that their effects follow an exponential distribution. We quantified the total number of beneficial mutations and their effects on resistance for an enzyme that is the main target of resistance against the antibiotic cefotaxime. Based on 48 unique beneficial mutations identified among more than 300 sequenced mutants, we estimate that at least 87 of the 2,583 possible point mutations (3.4%) in this enzyme are beneficial. We measured survival of these mutants on a range of antibiotic concentrations and derived estimates of resistance and fitness. The recovered distribution deviates from an exponential distribution due to the presence of a few mutants with a very large effect. The existence of such mutations will increase the predictability of adaptation and shorten the length of adaptive pathways.

## Introduction

Adaptation of asexual organisms to their environment is driven by the successive fixation of mutations that increase fitness. The effect-size of beneficial mutations varies and the distribution of beneficial fitness effects (DBFE) influences dynamics, outcome and repeatability of adaptation [1]–[5]. Nevertheless, we know little about the relative number and fitness effects of beneficial mutations for two main reasons. First, beneficial mutations are relatively scarce, which complicates empirical studies, because large numbers of beneficial mutations are required to accurately estimate the DBFE. Second, we lack a general theoretical basis for understanding DBFEs [6], [7]. Extreme value theory (EVT) provides predictions for adaptation to small environmental changes. Under these circumstances, the wild type has a relatively high fitness and beneficial mutations are then drawn from the tail of the distribution. Many commonly encountered parent distributions (including normal, gamma, exponential and logistic distribution) belong to the Gumbel domain. The tails of these distributions are all exponential, comprising many mutations of small effect and few of large effect [6]–[8]. Models that incorporate the effects of beneficial mutations therefore often assume this distribution to be exponential [3], [6]. The prediction of an exponential DBFE has been confirmed empirically using genotypes with high fitness [9]–[11], although one study has found a gamma distribution [12]. For low-fitness genotypes we lack a quantitative prediction, as often EVT can no longer be applied. The DBFE appears no longer exponential under these conditions and has been described by distributions which are approximately normal [10], [13].

Besides the Gumbel domain, two other domains of attraction exist which encompass classes of distributions with truncated tails (Weibull domain) and with heavy tails that decay as a power law (Fréchet domain) [7], [14]. Note that the differences between the three domains involve differences in the shape of the distribution and do not imply differences in absolute selection coefficients. While deviations from the Gumbel domain have been considered as biologically less likely [7], there is no fundamental reason why they may not occur [14], and it is therefore questionable whether the DBFE can be described by a general (exponential) shape. Two studies have already observed distributions with truncated tails [15], [16], but no support is known to us for Fréchet-type distributions. On average, adaptive walks to local fitness maxima are longer in the Weibull than in the Gumbel domain [17]–[19], while the likelihood for parallel evolution is reduced [14], [20]. In the Fréchet domain, the probability that the fittest alleles are fixed is enhanced, which brings the adaptive process close to the “greedy” limit [17], [18], [21] and increases the repeatability of adaptation [14], [18]. In addition, a heavy-tailed distribution reduces the fixation time of beneficial mutations, thereby mitigating the effects of competition among beneficial mutations (clonal interference) and reducing the opportunity for competition among clones carrying multiple mutations [3], [22], [23].

We study the number and properties of adaptive mutations in a single protein. So far, empirical studies of the DBFE were either based on relatively small sets of characterized mutations [9]–[12], [16] or large sets of uncharacterized mutations [13], [24]. We use TEM β-lactamase as a model system and study the effects of unique beneficial mutations for adaptation to cefotaxime (Ctx). Whereas the original TEM-1 enzyme hydrolyzes several β-lactam antibiotics effectively, it has promiscuous (i.e. low) activity towards Ctx. Several studies have shown that mutants with increased resistance to Ctx are found in nature [25] or can be selected by using iterative rounds of random mutagenesis and artificial selection [2], [26], [27]. These studies already identified several beneficial mutations and highly resistant mutants with multiple mutations, but none have characterized the full adaptive potential or the shape of the DBFE.

We use PCR mutagenesis to introduce random mutations into TEM-1 and select beneficial mutations at low Ctx concentrations. This way, we identify 48 unique beneficial mutations within a set of 864 selected isolates. The total number of possible beneficial mutations will be higher, and we estimate that at least 3.4% of all 2,583 possible base-pair mutations in the coding region of TEM-1 are beneficial. For each mutant, we measure Ctx resistance and infer its fitness at various Ctx concentrations by modeling survival and reproduction as a branching process. The distribution of resistance phenotypes belongs to the Fréchet domain and is characterized by a large number of small-effect mutants and a few exceptionally fit mutants. The DBFE is dependent on the fitness of the wild type, and when wild-type fitness is low (at higher antibiotic concentrations) the DBFE also belongs to the Fréchet domain.

## Results

### Estimating the total number of beneficial mutants

**Figure 1:**
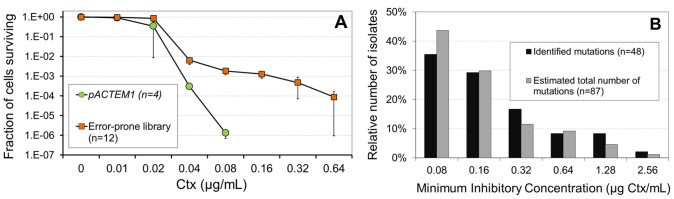
Survival of *E. coli* cells and distribution of the minimum inhibitory concentrations of Ctx. (A) Survival of *E. coli* cells with plasmid-borne TEM β-lactamase at various Ctx concentrations. Ctx concentrations are plotted on a log_2_ scale. Survival is plotted as the fraction of surviving cells (± S.D.) on a log_10_ scale. Comparison between cells carrying pACTEM1 plasmids and plasmids from the libraries generated by error-prone PCR. On average, 0.6 errors were introduced per amplicon. (B) Distribution of the Minimum inhibitory concentrations (MIC) of Ctx for isolates with a unique beneficial mutation in TEM (*n* = 48), and the corrected distribution which takes into account the estimated total number of beneficial mutations (*n* = 87; see Table 1). Ctx concentrations are plotted on a log_2_ scale.

*Escherichia coli* cells that harbor plasmid-borne TEM-1 have promiscuous activity towards Ctx. We introduced random mutations into TEM-1 by PCR mutagenesis and selected resistant bacteria at the lowest Ctx concentration (0.04 µg/mL) that showed differentiation in survival between wild-type and mutant libraries to minimize isolation bias against small-effect mutations (Figure 1A). In total, 864 isolates - 72 isolates for each of the 12 independent PCRs - were collected. As a first screen of the variation in Ctx resistance we determined the minimal inhibitory Ctx concentration (MIC) for each isolate. MIC values of the isolates ranged from 0.08 to more than 2.56 µg/mL, partly showing overlap with the MIC of the wild type (Figure S1). The distribution of MIC values is affected by the presence of isolates with the TEM-1 allele (which have reduced, but positive survival probability at 0.04 µg Ctx/mL), by spontaneous resistance mutations in the bacterial chromosome, by alleles containing multiple mutations and by the mutational bias of the DNA polymerase used for mutagenesis (Table S1). To avoid all these possible sources of bias, we focus on unique TEM mutants to determine the DBFE.

We used the collection of 864 isolates to estimate the total number of beneficial base-pair substitutions in three steps. First, we sequenced the TEM alleles of 310 isolates, while balancing the effort across the six MIC categories (Table S2). Second, we found 52 unique single mutants and determined which had a significant benefit by estimating their effect on Ctx resistance after transfer of the mutant allele to a naïve vector and host (i.e. taken from untreated frozen stocks). A sensitive continuous measure of Ctx resistance was obtained by estimating the Ctx concentration where a fraction 10^−4^ of the cells produce a visible colony, called the Inhibitory Concentration killing 99.99% (IC99.99, Figure S2). Using two-tailed *t*-tests and serial-Bonferroni correction, we found that the benefit of each of the 48 mutants with an IC99.99 higher than that of TEM-1 was significant (Table S2). Third, we tried to estimate the total number of beneficial mutations. Based on the number of unique beneficial mutations relative to the number of sequenced isolates in each MIC category, one can generate a maximum likelihood (ML) estimate of the total number of beneficial mutations per MIC category together with a 95% confidence interval (Table 1; see Material and Methods). When also taking the observed mutational bias of the polymerase into account (Table S1), a total of at least 87 beneficial mutations (95% CI 75–189) are estimated to exist. The ML estimates suggest that the highest MIC categories (1.28 and ≥2.56) have been sampled exhaustively, while our record is incomplete for the lowest MIC categories. Correcting for missing mutations therefore shifts the distribution of MIC values towards small-effect mutations (Figure 1B). Note that the applied procedure yields a conservative estimate of the total number of beneficial mutations due to possible variation in observation probabilities among mutants (see Material and Methods).

**Table 1:** Estimated number of beneficial mutations within each MIC category.

MIC category	Sequenced	Unique sequences	ML estimate	95% confidence interval	Unique mutations	ML estimate corrected for overlap	95% confidence interval	ML estimate corrected for mutation bias	95% confidence interval
0.08	29	18	27	21–51	17	31	22–74	38	28–116
0.16	35	15	17	15–23	14	15	14–21	26	24–34
0.32	36	12	12	12–15	8	8	8–11	10	10–12
0.64	36	7	7	7	4	4	4–7	8	8–18
1.28	36	5	5	5	4	4	4	4	4–8
≥2.56	36	1	1	1	1	1	1	1	1
**Total**	**208**	**58**	**69**	**66–160**	**48**	**63**	**53–118**	**87**	**75–189**

ML estimates and their 95% confidence intervals are based on the observed number of unique mutations relative to the sample size. Two corrections are introduced. (1) Some mutations are identified in multiple MIC categories and are subtracted from the category in which they are most rare (see Table S2 for details). (2) The error-prone polymerase displays mutation bias (see Table S1). Rare transversions are underrepresented and we extrapolated the estimates for the common transitions and transversions.

### Characteristics of the mutations

**Figure 2:**
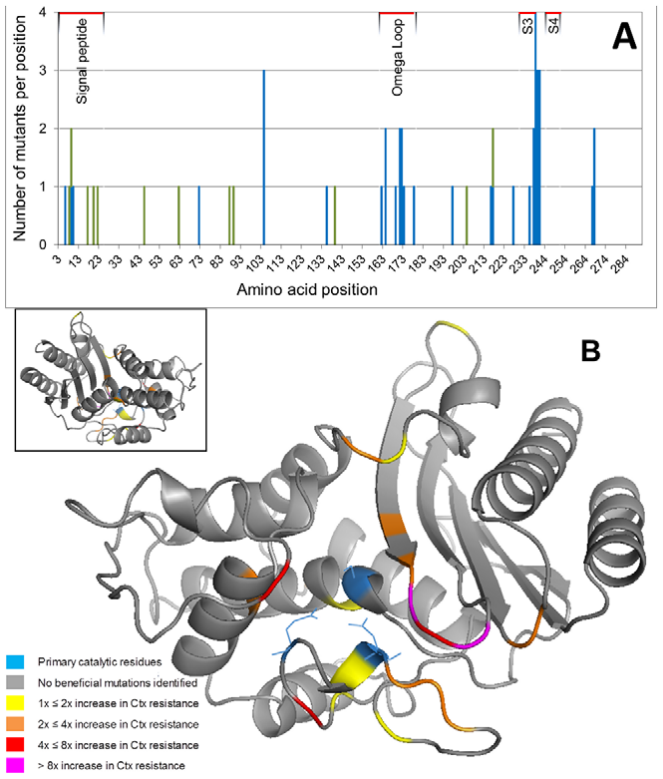
Number of observed beneficial mutations per codon and the effect sizes of the beneficial replacements. (A) Number of observed beneficial mutations per codon. Note that the numbering is based on the consensus sequence of class A β-lactamase and that codons 1, 2, 239 and 253 are not present in TEM. Green bars represent synonymous mutations and blue bars non-synonymous mutations. (B) The effect sizes of the beneficial replacements are mapped onto the crystal structure of TEM-1. Colours indicate the increase in Ctx resistance relative to TEM-1 caused by the mutation with the largest effect size at each position. The insert shows the same structure rotated horizontally by 180°.

The 48 identified beneficial mutations were located at 32 of the 291 amino acid positions (Figure 2A). The TEM polypeptide is composed of a signal peptide and a mature protein. The majority of the mutations (*n* = 41) were located in the mature protein, while seven mutations were located in the signal peptide. Remarkably, 10 of the 48 mutations are synonymous mutations. Mutations in the mature protein (average IC99.99 = 0.160 µg Ctx/mL) had a significantly larger effect on resistance than those in the signal peptide (IC99.99 = 0.078 µg Ctx/mL; two-sample *t* = 3.520, df = 46, two-tailed *P* = 0.0010), and non-synonymous mutations (IC99.99 = 0.166 µg Ctx/mL) had a significantly larger effect than synonymous mutations (IC99.99 = 0.081 µg Ctx/mL; *t* = 3.238, df = 46, two-tailed *P* = 0.0022). Several codons gave rise to multiple beneficial mutations. Particularly the codons E104, G238, E240, and R241 stood out, where three-four of the five-six accessible amino acid replacements increased Ctx resistance. The average resistance improvement per codon depended positively on the number of adaptive mutations observed per codon (*F*_1,46_ = 26.968, *P*<0.001).

The clustering of mutations becomes even more evident from their position in the tertiary structure of the mature protein (Figure 2B). The catalytic serine residue (S70) is positioned between a domain consisting of five β-sheets packed against three α-helices and a domain that consists of eight α-helices. Except for E197V and A227T, all amino acid replacements are located in the oxyanion pocket, which surrounds the catalytic site, or on loops near the entrance of this pocket. The majority of the mutations are located in the Ω-loop and the hinge between the β-sheets S3 and S4, which are located directly opposite to each other and harbor 10 and 13 mutations, respectively. Eighteen of the non-synonymous mutations have to our knowledge never been identified, whereas twenty were previously encountered in clinical isolates or directed evolution experiments (Table S2; reviewed in [25]). The set of new mutations includes mainly small-effect mutations, but includes also R241P, which ranks third in terms of effect size. Three of the 10 synonymous mutations have been found before in clinical isolates (Table S2).

### Distribution of resistance effects

The 48 beneficial mutants showed substantial variation in Ctx resistance (IC99.99). Resistance ranged from 0.055 to 1.410 µg Ctx/mL, where TEM-1's resistance is 0.052 [SD = 0.0010] µg Ctx/mL. However, TEM-1's resistance is still significantly higher than that of bacteria carrying the same plasmid without TEM-1 (IC99.99 = 0.033 [SD = 0.0034] µg Ctx/mL; two-sample *t* = 8.627, df = 7, two-tailed *P*<0.0001), indicating that deleterious mutations are possible as well.

**Figure 3:**
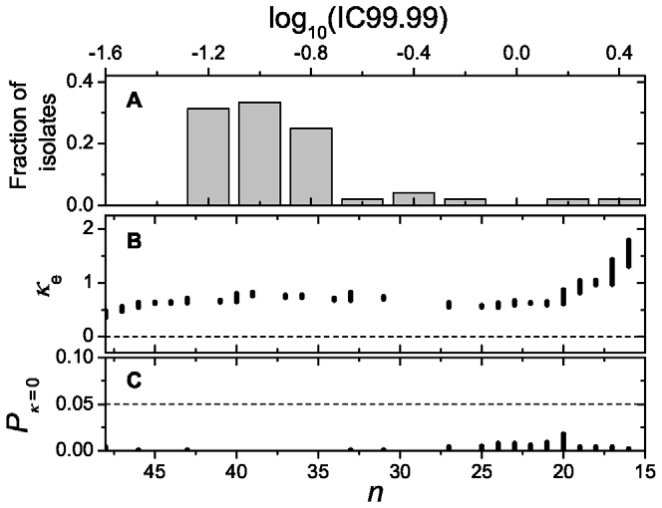
Distribution and likelihood analysis. (A) Distribution of Ctx resistance levels (IC99.99) of 48 beneficial mutants. (B) Likelihood analysis of the estimated shape parameter (*κ*_e_). *κ*_e_ is plotted against the number of beneficial mutants (ranked by their effect) that were included for the estimation (*n*) by using a fitness threshold (*w*_c_). Because each value of *n* corresponds to a range of values for *w*_c_, there is also a range of *κ*_e_ associated with every *n*. The dashed line corresponds to *κ* = 0. *κ*_e_ is well above zero in the whole depicted range, suggesting that the distribution belongs to the Fréchet domain. (**C**) The *P*-value corresponding to the hypothesis *κ* = 0 is plotted against *n*. For all choices of *w*_c_ and *n*>14 the null hypothesis can be rejected with more than 95% confidence as indicated by the dashed line.

To determine the shape of the distribution of resistance effects of beneficial mutations (Figure 3A), we fitted the data to a generalized Pareto distribution (GPD). The GPD is a right-skewed distribution, parameterized with a scale parameter *τ* and a shape parameter *κ*, that can model tails of a wide variety of distributions. The shape parameter determines whether the tail distribution belongs to the domain of distributions with exponential tails (*κ* = 0; Gumbel domain), truncated tails (*κ*<0; Weibull domain) or heavy tails that decay as a power law (*κ*>0; Fréchet domain). Our estimates of *κ* (*κ*_e_) are typically between 0.5 and 1 (Figure 3B), indicating that the distribution belongs to the Fréchet domain. EVT can only be applied when the observed values are drawn from the tail of the distribution. This can be verified by gradually removing mutations at the left side of the tail; when *κ*_e_ does not change during this procedure this is evidence that the DBFE corresponds to the tail of some distribution. The estimate *κ*_e_ is roughly constant when considering the fittest 48 to 20 mutations (Figure 3B and 3C), and becomes unstable beyond this point due to the small number of data points. A likelihood-ratio test shows that *κ*_e_ differs from the Gumbel domain hypothesis (*κ = 0*) with more than 95% confidence for *n*>15 (Figure 3C).

We checked whether the results are robust with regard to the criterion used to calculate resistance levels and depended on the presence of specific mutations. Using the same analysis for the Ctx concentration where a fraction 10^−2^, 10^−3^ and 10^−5^ of the cells survive, we confirmed that *κ*_e_ was in the Fréchet domain irrespective of which fraction was used to calculate resistance levels (Figure S3). The ML estimator for the GPD is known to be sensitive to individual mutations with exceptionally high resistance levels [28]. In our dataset, the G238S mutant is much fitter than any other mutation, and we reran the analysis without this mutant (Figure S4). Although *κ*_e_ is lower without G238S it is still in the Fréchet domain. Finally, we also estimated *κ* while including only the non-synonymous mutations in the mature protein for analysis, and again confirmed the rejection of Gumbel in favor of the Fréchet domain (Figure S5). The number of synonymous mutations was too low to perform a similar analysis of this subset.

### Distribution of fitness effects

The fraction of cells that grow into a visible colony (*P*_sur_) at each Ctx concentration (used to estimate IC99.99) can also be used to estimate the fitness effects of the 48 beneficial mutants in the presence of Ctx. By simulating reproduction and survival as a branching process, we can estimate the probability that an individual cell survives onto the next generation (*p*) from *P*_sur_ (see Material and Methods). When the cell survives into the next generation it will divide and the expected number of offspring is 2*p*, which is equivalent to the Wrightian fitness *W*. The selection coefficient *s* is then calculated by *W*_i_/*W*_0_–1, where *W*_i_ and *W*_0_ are the fitness of mutant and wild type, respectively. Because we assume no effects of the antibiotic on the birth rate (generation time), *W* has an upper bound of 2, and the selection coefficient is restricted to values between 0 and 1.

**Figure 4:**
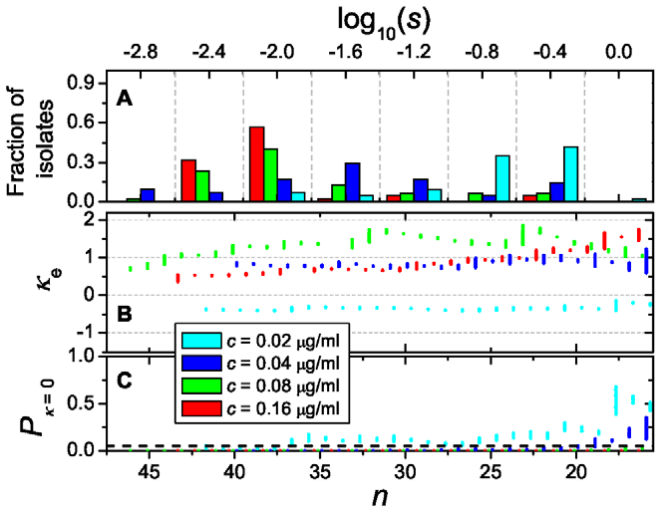
Distribution of the selection coefficients, likelihood analysis, and *P*-value. (A) Distribution of the selection coefficients (*s*) of the 48 beneficial mutations for four low Ctx concentrations as inferred from survival data using a branching model. Vertical dashed lines show the boundaries between the logarithmic bins used in the analysis. The distribution shifts towards smaller values of *s* as the antibiotic concentration increases, while a group of very fit mutants persists near the maximum selection coefficient *s* = 1. (B) Likelihood analysis of the estimated shape parameter (*κ*_e_) for the four distributions. The ML estimate *κ*_e_ is plotted against the number of beneficial mutants that were included for estimation (*n*) by using a fitness threshold (*w*_c_). Because each value of *n* corresponds to a range of values for *w*_c_, there is also a range of *κ*_e_ associated with every *n*. (C) The *P*-value corresponding to the hypothesis *κ* = 0 is plotted against *n*. The null hypothesis can be rejected with more than 95% confidence for data points beneath the dashed line. All panels correspond to *N*_lim_ = 100,000 bacteria and *T*_exp_ = 40 generations. See Figure S7 for a similar analysis, where *T*_exp_ = 40 generations and *N*_lim_ = 1,024 bacteria.

In principle, we can only determine selection coefficients under conditions that allow some survival of the wild type (i.e. up to 0.08 µg Ctx/mL). In order to extend our analyses to higher concentrations, we instead calculate selection coefficients with respect to the least fit beneficial mutant at 0.02, 0.04, 0.08 and 0.16 µg Ctx/mL. The estimates of *κ* for the distribution of selection coefficients (Figure 4A) suggest that the shape of the distribution depends on the Ctx concentration: at 0.02 µg/mL, *κ*_e_ is negative, suggesting a truncated tail (Weibull domain) – although it cannot be distinguished statistically from 0 (Gumbel domain), while at concentrations of 0.04 µg/mL and higher *κ_e_* is significantly positive, suggesting a heavy tail (Fréchet domain; Figure 4B and 4C). Our interpretation of these results is that at low Ctx concentration, the DBFE is affected by the upper limit of 1 set for *s*, and only at higher Ctx concentrations it can reveal its unconstrained shape. Since other studies have used *P*_sur_ itself as proxy for fitness, we have also looked at its distribution (Figure S6), more precisely at the DBFE corresponding to 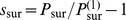, where 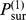 is the observation probability of the least fit beneficial mutant observed. This estimator yields very large selection coefficients, but also supports Fréchet for Ctx concentrations higher than 0.04 µg/mL.

## Discussion

We studied the adaptive potential of the enzyme TEM-1 β-lactamase in a novel environment. We generated random mutations, estimated the total number of beneficial mutations and determined the distribution of their effects on Ctx resistance and fitness. We characterized 48 beneficial mutations and our statistical analyses suggest that at least 3.4% of all mutations are beneficial under the experimental conditions, a substantial fraction of which are synonymous mutations. We also found that the distribution of resistance and fitness effects of these mutations belongs to the Fréchet domain of heavy-tailed distributions.

Although the adaptive potential that we estimate for TEM-1 β-lactamase is high, the real number of beneficial mutations is likely to be even higher, because our estimates are conservative. Other recent studies have also found frequencies of beneficial mutations in the order of 1–10% [3], [12], [24], [29], [30]. One should take into account that TEM is a known target for Ctx resistance [2], [26], [27] and already has promiscuous activity towards this antibiotic, which is an important prerequisite for successful directed evolution [31]. During the process of directed evolution, iterative rounds of mutagenesis and artificial selection are performed. The difference with our study is mainly that we are interested in characterizing the full adaptive potential of the enzyme, including beneficial mutations with small effect, whereas directed evolution studies are biased towards finding large-effect mutations, as well as functional combinations of mutations. As a consequence, 18 of the 38 non-synonymous mutations that we identified are new, including mutations with large effect (see Table S2). Protocols for directed evolution sometimes allow the simultaneous selection of multiple mutations. These protocols have been applied to TEM as well [2], [26], [27], but it is then unclear whether the identified mutations are beneficial in the wild-type background or only when particular other mutations are present. With each fixation the mutational neighborhood changes, thereby granting access to mutations that are inaccessible for the original background [32] and obstructing access to others due to sign epistatic interactions [2], [4]. This is for example true for compensatory mutations such as M182T and T265M, which restore stability to enzymes that have become unstable as a result of mutations that increased activity [33]–[35], but are not beneficial in the stable wild-type background. Although epistasis affects the identity of mutations which are beneficial with each change in the genetic background, the DBFE may be unaffected [36]. This is an important topic for future investigation.

Beneficial mutations that increase Ctx resistance either increase the concentration of correctly folded enzyme or the activity per molecule, and may affect both aspects in opposite ways [35], [37]. Mutations in the signal peptide and synonymous mutations will likely increase enzyme levels. For example, mutation Q6R is known to increase periplasmic expression levels of TEM [38]. Synonymous mutations may prolong mRNA half-life [39], facilitate correct folding and export of the protein [40], or affect mRNA stability and translation [41]. Interestingly, Lind *et al.* [42] showed that the distribution of fitness effect of deleterious mutations in ribosomal proteins did not differ for synonymous and non-synonymous mutations, while Lalic *et al.* [30] also identified multiple synonymous mutations among mutations that expanded the host range for a plant virus. We found that the shape of the distribution did not change significantly when excluding synonymous mutations, while synonymous mutations had on average a lower effect on Ctx resistance than non-synonymous mutations. Vakulenko *et al.* [43] showed that poor activity of TEM-1 is caused by steric hindrance due to the bulky side-groups of modern cephalosporins such as Ctx. The majority of the identified replacements were located in close vicinity to the catalytic site in the oxyanion cavity or on loops near the entrance of this cavity (Figure 2B). This suggests that they either have a specific interaction with the Ctx substrate or enlarge the cavity, as has been suggested for the mutations R164S and G238S [2], [43], [44].

We presented a novel procedure to infer fitness from the survival of bacterial cells at various Ctx concentrations. Previous studies made attempts to estimate fitness benefits of antibiotic resistance mutations using competition assays or growth rate measurements [10], [45], [46], but these measurements typically involve substantial efforts and large measurement errors (but cf. [42]). We quantified the probability that a single cell produces a visible bacterial colony by simulating reproduction and survival per cell generation as a branching process. The advantage of this procedure is that each surviving colony contributes to an aggregate estimate of the survival probability per cell generation. A potential limitation of the current approach is the assumption that fitness is only affected by differences in survival and not by differences in birth rate (i.e. generation time). Our assumption is supported by Negri *et al.* [45], who observed that initial growth rates did not differ between different mutants of TEM. Nevertheless, it will be interesting to compare the fitness estimates yielded by our method to estimates from other methods, such as direct competitions, and to confirm the assumption underlying our procedure.

The results of our study combined with previous studies on the shape of the DBFE suggest that the DBFE cannot be generally described by an exponential distribution. Instead, its shape will vary among organisms and environments. Examples are now known for all universality domains (Gumbel, Weibull and Fréchet). Most studies have encountered an exponential DBFE [9]–[11], two studies have observed distributions with truncated tails [15], [16], while our study is the first to find that the effects of beneficial mutations follow a distribution that belongs to the Fréchet domain. Lalic *et al.* [30] recently reported a DBFE for an RNA plant virus that was best fitted by a Pareto distribution, a member of the Fréchet EVT class. However, their distribution was based on the effects of 20 random mutations and may have included deleterious ones, while their inferred estimate of shape parameter *κ* was very small (*κ*≈0.045), making it unlikely that the Gumbel domain could be rejected with statistical confidence. Also in our case, the shape of the distribution depends on environmental conditions: we observed heavy tailed distributions at high Ctx concentrations, but found a truncated or exponential tail at the lowest Ctx concentration where wild-type fitness was high. MacLean and Buckling [10] also found the DBFE to depend on the wild-type fitness. They tested the DBFE of 15 beneficial mutants in *rpoB* at various rifampicin concentrations and could reject the exponential null hypothesis at high concentrations. However, they did not estimate the shape parameter *κ* of the GPD.

An organizing principle that may help explain the variation in observed DBFEs is the variation in pleiotropic constraints associated with the beneficial mutations studied. Mutations that increase resistance to an antibiotic also incur fitness costs due to pleiotropic effects on other cellular functions, which often make their benefit conditional on the presence of the antibiotic [47]. The enzyme we chose for our study may be atypical in this respect, since the only catalytic activity of β-lactamases involves the hydrolysis of β-lactams [48], while resistance to other antibiotics is often caused by changing structural or metabolic components that are targeted by the antibiotic [47]. It is conceivable that the pleiotropic constraints experienced during adaptation of a specialized enzyme are different from those involved in adapting a component with an existing function, although it is less clear in what respect. Limited pleiotropic constraints are consistent with the presence of a few exceptionally fit mutants in our system and the support we found for a Fréchet-type distribution. However, it may be naïve to think that pleiotropic constraints are smaller for our enzyme, because both internal constraints from counteracting effects on enzyme activity and stability, and external constraints through negative effects from high concentrations of periplasmic enzymes interfering with nutrient uptake and from protein aggregates and the associated recruitment of limiting chaperones may occur [35], [37].

Our observation that beneficial mutations follow a Fréchet distribution has implications for the repeatability and dynamics of adaptation, in particular in the context of antibiotic resistance [47]. Fréchet distributions are characterized by many small-effect mutations and a “heavy tail” with a few mutations with exceptionally large effects. The existence of these latter mutations increases the short-term probability of parallel evolution, which can be quantified by the probability *P*_2_ *(n)* that two replicate populations fix the same mutation out of *n* available beneficial mutations. When the DBFE is in the Gumbel domain, *P*_2_(*n*) = 2/(*n*+1), which is about twice the neutral value [20]. With a moderate departure from the Gumbel into the Fréchet domain, *P*_2_(*n*) is still proportional to 1/*n*, but with a coefficient that increases with increasing *κ* and diverges at *κ* = ½, where the distribution ceases to have a finite second moment [14]. For ½<*κ*<1, the probability of parallel evolution decreases more slowly with the number of neighboring genotypes as 1/*n* ^−(1/*κ*−1)^ [J. Krug, unpublished], while adaptation becomes even more deterministic for *κ*>1, when *P*_2_(*n*) approaches a nonzero constant *P*_2_ = 1−1/*κ* for large *n* [J. Krug, unpublished; [49]]. Similarly, the probability *P*_max_ that the mutation of largest effect is fixed approaches a constant value that is bounded from below by *P*_2_ [J. Krug, unpublished; [49]]. To illustrate this, the estimates of *P*_2_ and *P*_max_ were derived from the experimentally determined selection coefficients. Both measures of repeatability increase monotonically with increasing Ctx concentration, and for concentrations above 0.04 µg Ctx/mL repeatability is much greater than predicted for the Gumbel class (Table S3 and Figure S8). These results are, at least qualitatively, consistent with a recent observation that 10 of the 12 replicate lines of TEM-1 β-lactamase adapting to Ctx substituted G238S, the largest-effect mutation of our set of 48, as the first mutation [2], as well as with a report of remarkable parallel evolution towards trimethoprim resistance [1].

The shape of the DBFE also affects the length of adaptive walks. Assuming a maximally rugged, uncorrelated fitness landscape, recent theoretical studies have shown that the average number of adaptive steps required to reach a local fitness maximum increases logarithmically with initial fitness rank when *κ*<1, but becomes independent of this quantity when *κ*>1 [17]–[19]. In the extreme Fréchet domain (*κ*>1), the mutation of highest fitness is overwhelmingly likely to be fixed in each step, so the walk effectively becomes ‘greedy’ and typically terminates after a small number of substitutions [21]. A heavy-tailed DBFE thus provides a possible explanation that could account for the short adaptive walks observed in recent experiments [50], [51]. Finally, the very fit mutants that are in the broad tail of the distribution can swiftly sweep to fixation and outcompete mutants with smaller effect. This mitigates the effects of competition among beneficial mutations (clonal interference), and particularly decreases the probability that multiple beneficial mutations accumulate on the same genetic background during a selective sweep [3], [22], [23]. Given these diverse and fundamental consequences, it is imperative to understand the conditions that yield Fréchet-type DBFEs.

## Materials and Methods

### Bacterial strain and plasmids

*Escherichia coli* strain DH5*α*E was used as the host for all plasmids. TEM-1 was amplified from pBR322 and cloned into the pACSE3 plasmid to yield pACTEM1 [26]. This plasmid carries a tetracycline resistance gene; we added 15 µg tetracycline/mL in all cultures to ensure its preservation. Cultures were grown at 37°C. Expression of TEM alleles is controlled by the *pTac* promoter that is in turn regulated by the lac repressor which is encoded by the *lacI* gene on pACSE3. Expression of TEM alleles was induced by adding 50 µM isopropyl-β-D-thiogalactopyranoside (IPTG) [26].

### Mutagenesis

We introduced random mutations into TEM-1 using the GeneMorph II random mutagenesis kit (Stratagene) and the primers P3 (TCATCCGGCTCGTATAATGTGTGGA) and P4 (ACTCTCTTCCGGGCGCTATCAT), which flank the multiple cloning site of pACSE3. The mutagenesis conditions were modified to induce ∼0.6 mutations per amplicon by using 100 ng of template and replacing one twentieth of Mutazyme II polymerase by *Pfu* polymerase (Stratagene). The cycling program consisted of: denaturation at 95°C for 2 min, 30 cycles of denaturation (30 sec at 95°C), annealing (30 sec at 60°C), and extension (75 sec at 72°C), followed by a final step at 72°C for 10 min. Substitutions that arise early during the PCR reaction dominate the final mixture due to the branching nature of the amplification process [52]. To increase the diversity of mutations, we carried out twelve independent PCRs.

The immediate mutational neighborhood (i.e. Hamming distance = 1) of TEM-1 contains 2,583 point mutants (excluding insertions and deletions). Given the average observed mutational load of ∼0.6 errors per sequence (based on sequencing 24 non-selected transformants) and ∼10^6^ transformed cells per library, one expects that all 2,583 one-step mutants are present in the transformation mixture at least once (http://guinevere.otago.ac.nz/cgi-bin/aef/pedel.pl, [53]), but that a significant fraction of the amplicons contains no or multiple mutations.

### Isolation of mutants with increased Ctx resistance

Amplicons were purified using the GenElute PCR Clean-Up Kit (Sigma-Aldrich) according to the manufacturer's instructions. Amplicons were digested with *BspHI* and *SacI* restriction enzymes (New England Biolabs), purified, ligated into pACSE3 using T4 DNA ligase (New England Biolabs), and then electroporated into DH5*α*E. The original pACTEM1 plasmid was also electroporated into DH5*α*E. Cells were allowed to recover in SOC medium (20 g tryptone and 5 g yeast extract/liter supplied with 10 mM NaCl, 2.5 mM KCl, 10 mM MgSO_4_, 10 mM MgCl_2_, and 20 mM glucose) for 60 minutes.

To select mutants with increased resistance to Ctx (Sigma-Aldrich) relative to TEM-1, we first determined at which Ctx concentration pACTEM1 (*n* = 4) and the mutated libraries (*n* = 12) produce viable colonies. Directly after recovery, three aliquots from each transformation mixture were spread onto LB agar plates without Ctx - to determine the number of transformants per library - and onto LB agar plates with a two-fold increase of Ctx concentrations ranging from 0.01 to 0.64 µg/mL. Plates were incubated (40 h) and colony counts revealed the library size and the surviving fraction at each Ctx concentration. The lowest concentration at which pACTEM1 clearly displayed reduced survival was 0.04 µg Ctx/mL (Figure 1). For all 12 libraries, we subsequently picked a random sample of 72 colonies from plates with 0.04 µg Ctx/mL. This adds up to a total of 864 clones, which were grown overnight (O/N) in microtiter plates with LB medium. Ten-% glycerol stocks of all isolates were stored at −80°C.

### Pre-screening by MIC assay

To determine which Ctx resistance classes were present among the isolates we first screened all isolates by measuring the minimum inhibitory concentration (MIC) using the broth dilution method. Before screening, cultures were revived by diluting them 1∶100 in LB medium with tetracycline and grown O/N. Cultures were then rediluted at a titer of ∼10^5^ cells/mL into fresh LB medium in a series of microtiter plates with a two-fold increase in Ctx concentrations ranging from 0.01 to 2.56 µg/mL. Cultures were incubated and the MIC was established as the lowest Ctx concentration that gave no visible growth. Note that spontaneous mutations outside the TEM-1 gene may have occurred during the selection procedure. These were not excluded before the MIC assay, and may partly explain the presence of survivors with the wild-type allele at high Ctx concentrations.

### Sequencing and mapping of mutations

To identify mutations in TEM, we amplified this gene using *Pfu* polymerase and the primer pair P3–P4. PCR products were sequenced using the BigDye sequencing kit. To obtain a balanced sample, we divided all isolates into six classes according to their MIC (0.08, 0.16, 0.32, 0.64, 1.28 and ≥2.56 µg Ctx/mL). We continued sequencing until we identified 36 sequences with a single substitution in each MIC class, balanced across the 12 replicate PCR reactions. Double mutants and wild-type (TEM-1) sequences were discarded from further study. In addition, we sequenced ten colonies from the pACTEM1 libraries which survived on the plates with 0.08 µg Ctx/mL to examine the presence of spontaneous mutations in TEM-1, which were not present. Sequences were analyzed using MEGA software. Mutations were numbered according to Ambler *et al.* [54]. Amino acids replacements were mapped onto the crystal structure of TEM-1 in PyMOL [55] using the coordinates of the wild-type structure (Protein Data Bank ID: 1ZG4).

### Resistance assay

Ctx resistance levels of all unique mutants were estimated by determining survival on agar plates in the presence of Ctx. To exclude spontaneous mutations in the *E. coli* background or the plasmid (outside TEM), we ligated the sequenced PCR products back into pACSE3 and electroporated the plasmids into a new batch of DH5*α*E. For each mutant, two to four replicate cultures were plated at the appropriate dilutions onto a series of LB agar plates with two-fold increases in Ctx concentrations ranging from 0.01 to 5.12 µg/mL. After 40 h of incubation, colony counts were compared to the effective library size to determine the fraction of cells that produced a visible colony at each Ctx concentration. The resistance level (IC99.99) was defined as the Ctx concentration at which a fraction 10^−4^ of the cells produces a visible colony. This value was calculated by linear interpolation from the two adjacent data points. The threshold of 10^−4^ cells was chosen based on the abundant occurrence of chromosomal resistance mutations in *OmpF*, which are known to occur at a frequency of 10^−5^ [45], [56]. The MIC and IC99.99 values of the 52 isolated mutants with a single mutation were a strongly correlated (*r* = 0.90, n = 50, *P*<0.001).

### Statistical procedures

We estimated how many mutants have been overlooked in our isolation procedure by adapting the procedure described in [57]. When all mutants have equal observation probabilities, the probability to observe *n* unique mutants from a total of *N* existing mutants in *k* picks, *P_N_* [*X_k_* = *n*], can be calculated analytically. Since *k* and *n* are obtained from the experiments, one can calculate the most likely value of *N* by means of a maximum likelihood (ML) analysis by demanding *P_N_* [*X_k_* = *n*] to be maximal at the most likely *N*, *N*_ML_. The associated 95% confidence interval is obtained by calculating the values of *N*_ci_ for which the sum over the probabilities 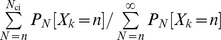 is smaller or larger than 0.025 and 0.975, respectively. Given the potential differences in detection probability between large and small-effect mutations, we ran a separate analysis for the six MIC classes. By assuming equal observation probabilities for all mutants within a MIC class and by isolating three transformants per MIC category from the same library, we may introduce a systematic error that increases the number of identical mutants; therefore, our estimate of the total number of mutants is conservative.

We tested whether the 52 unique mutants had a significantly higher resistance phenotype (IC99.99) than TEM-1 using two-sample two-tailed *t*-tests. This leaves the possibility open for mutants that are less resistant than TEM-1, of which we found four (Table S2). Since the variance and mean of our IC99.99 estimates were strongly positively correlated (*r* = 0.904), all *t*-tests and regressions were performed using log(IC99.99), which homogenized the variance and removed the positive correlation (*r* = −0.030).

### Generalized Pareto distribution

To assign the shape of the DBFE to a domain of extreme value distributions, we fitted a generalized Pareto distribution (GPD) to the obtained data, using a maximum likelihood (ML) procedure, essentially following the procedure in [58]. The GPD is characterized by a scale parameter *τ* and a shape parameter *κ*. The fit yields an estimate for *κ* (*κ*_e_), whose value attributes the distribution to one of three universality domains [59]. The GPD is given by:
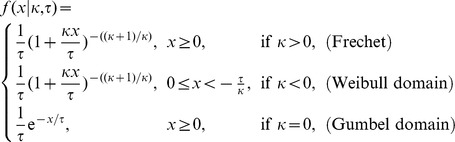
The GPD describes the limiting tail behavior, which is expected to be visible above a certain threshold (*w*_c_). If the data correspond to the tail of some distribution, sliding the threshold shifts the scale parameter by 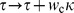, but leaves the estimate for *κ* unaffected. Although the wild-type fitness seems a “natural” threshold, a higher threshold is sometimes preferable. For example, Beisel *et al.* [58] proposed to use the fitness of the beneficial mutant with the smallest effect as *w*_c_. This was done to remove the error that arises from overlooking small-effect mutations and to allow the analysis of gain-of-function mutations. Threshold selection is a standing problem for this type of analysis and different approaches exist (see [60], [61]). In the present context, a key concern is that the probability of overlooking mutations depends on the effect-size. We tackled this problem by estimating *κ* for various choices of *w*_c_. One should then obtain a range of *w*_c_ values in which *κ_e_* is stable. If this is not the case, we are probably not observing the tail of a distribution in the sense of extreme value theory.

A likelihood ratio test is performed to assess whether the null hypothesis *κ* = 0 (Gumbel domain) [7] can be ruled out. Following the procedure in [58], we first calculated 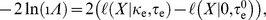 where 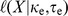 is the log-likelihood of the measured fitness values given *κ_e_* and *τ*_e_, and 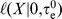 is the log-likelihood for estimating value 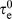 under the hypothesis that *κ* = 0. Next, we produced 1000 datasets for each value of *w_c_* by numerically picking random numbers from a GPD with parameters *κ* = 0 and 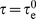. The fraction of cases for which −ln(*Λ*) is larger for the experimental data than for the numerically obtained data gives the *P*-value corresponding to the hypothesis *κ* = 0. We checked to what extent measurement error altered our estimates. In that case, the likelihood (*L*) is calculated by taking products of the convolutions of the probability density function with Gaussians representing the uncertainty in the measurement of the fitness values (see [58]): 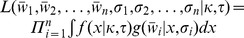, where *n* is the number of considered mutants, *f* is the GPD, and *g* is a Gaussian with mean *w_i_* and variance *σ_i_*. The values of *w_i_* and *σ_i_* are extracted from the replicated measurements of the IC99.99 or fitness. As the estimates for *κ* and *τ* change very little when taking into account measurement error, we only present data for the unperturbed cases.

### Survival probability as a fitness measure

By modeling reproduction and survival as a branching process, we established a relation between survival in the presence of the antibiotic and fitness. Suppose that subsequent generations of individuals in a population reproduce (double) themselves with probability *p* or die with probability (1−*p*). The expected number of offspring is then 2*p*, which can be identified as the Wrightian fitness *W*. In order to be visible, a colony of bacteria has to reach a minimal size, *N*_lim_≈100,000 bacteria in the time span the experiment is carried out, *T*_exp_≈40 generations. The probability that this happens is the measured survival probability *P*_sur_. In order to be able to infer the value *p*, and thus *W*, from the measured *P*_sur_, we simulated the branching process for 1000 values of *p*, equidistantly distributed between 0 and 1, and measured the fraction of runs in which *N*_lim_ was reached in 40 or less generations, *f*(*p*). If our model is correct, *P*_sur_ = *f*(*p*) and therefore *p* = *f* ^−1^(*P*_sur_). We calculated *p* by backtracking for which value the measured *P*_sur_ would be expected. Missing values of *f*(*p*) were obtained by linearly interpolating its logarithm. We then calculated the selection coefficient of the mutants with respect to the least fit beneficial mutant observed (*W*_1_), *s* = *W*/*W*_1_−1. Note that calculating *s* with respect to the wild-type fitness instead does not change the estimates considerably, but would not allow us to use the data corresponding to 0.16 µg Ctx/mL. We verified that our measurements do not depend too strongly on the particular choice of *N*_lim_ (see Figure S7).

By construction, *s* can only take values between 0 and 1 due to the restriction 2≥2*p*>1. At a first glance, it appears as if the sharp upper cutoff for *s* will inevitably force the distribution towards the Weibull domain. However, at 0.04, 0.08, and 0.16 µg Ctx/mL, where nearly all mutants have strongly reduced survival probabilities, the upper bound is not relevant and other classes of distributions are possible. At 0.02 µg Ctx/mL the recovered distribution is in the Weibull domain (albeit not significantly different from Gumbel), but note that here the DBFE may be affected by the upper limit for *s* of 1.

By means of the selection coefficients we further calculate the probabilities of parallel evolution, 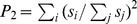, and the probability for the fittest mutant to take over the population, 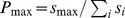, where *s*_max_ is the selection coefficient corresponding to the fittest mutant and the sums in the expressions run over all beneficial mutations [20]. Note that both measures correspond to the strong selection weak mutation regime and are obtained using Haldane's expression for the fixation probability, *π* = 2*s*, which presumes 2*s*«1 [62]. For larger values of *s*, the expressions would need to be modified, but the overall tendency of increasing *P*_2_ and *P*_max_ for increasing *κ* values is maintained.

## Supporting Information

Figure S1 Histogram displaying the relative frequencies of MIC values of *E. coli* cells with plasmid-borne TEM β-lactamase that carry (1) the wild-type TEM-1 allele, (2) alleles from the mutated libraries that survived exposure to 0.04 µg Ctx/mL on plates (*n* = 864), and (3) alleles from the mutated libraries that carry only a single mutation (based on sequencing a sample of 310 isolates). MIC values were determined without transformation into a fresh isogenic background, and the potential presence of mutations outside the TEM gene was not eliminated in this assay. Note that Ctx concentrations are plotted on a log_2_ scale.
(TIF)

Figure S2 Survival of *E. coli* cells with plasmid-borne TEM β-lactamase at various Ctx concentrations. Survival is plotted as the fraction of surviving cells (± S.D.) on a logarithmic scale, and Ctx concentrations are plotted on a log_2_ scale. Comparison between cells carrying pACTEM1 and three mutants with a large (G238S), intermediate (E104K) and small-effect beneficial mutation (I173V), which shows the range of resistance effects encountered in this study.
(TIF)

Figure S3 Likelihood analysis of the estimated shape parameter (*κ*_e_) of the GPD. In the upper panel, *κ*_e_ is plotted against the number of beneficial mutants, ranked by their effect on resistance, that were used for the estimation (*n*) by using a threshold (*w*_c_). The IC99.99 was calculated as the Ctx concentration at which a fraction of 10^−4^ cells survived. This figure displays *κ*_e_ corresponding to fractions of 10^−2^, 10^−3^, and 10^−5^ surviving cells to calculate the inhibitory Ctx concentration. Note that every *n* corresponds to a range of values of *w*_c_ and thus to a range of estimates for *κ*_e_. The dashed line corresponds to *κ = 0*. *κ*_e_ exceeds 0 for 15≤*n*≤50. The *P*-value corresponding to the hypothesis *κ = 0* is plotted against *n* in the lower panel. The dashed line corresponds to the 95% confidence level. The hypothesis can be rejected with more than 95% confidence for all choices of *w_c_* and 15≤*n*≤48.
(TIF)

Figure S4 Analogous to Figure S3. The results of the likelihood analysis without the fittest mutant (G238S) are shown. This mutant is excluded to determine whether the condition *κ*>0 does not solely depend on this mutation. Compared to Figure S3, *κ*_e_ shifts towards smaller values, but remains positive. The *P*-values obtained for the hypothesis *κ = 0* are generally larger compared to the analysis with G238S, but remain below 0.05 (shown by the dashed line) for 30≤*n*≤45.
(TIF)

Figure S5 Analogous to Figure S3. The results of the likelihood analysis for only the non-synonymous mutations in the mature protein (*n* = 35) are shown. The removal of synonymous mutations does not affect the shape of the distribution.
(TIF)

Figure S6 (A) Fractions of isolates are plotted versus the logarithm of the selection coefficient (as inferred from survival data) for four low Ctx concentrations. Vertical dashed lines show the boundaries between the logarithmic bins used in the analysis. (B) Likelihood analysis of the estimated shape parameter (*κ*_e_) for the distribution of selection coefficients. The ML estimate *κ*_e_ is plotted against the number of ranked beneficial mutants that were included for estimation (*n*) by using a fitness threshold (*w*_c_). Because each value of *n* corresponds to a range of values for *w*_c_, there is also a range of *κ*_e_ associated with every *n*. (C) The *P*-value corresponding to the hypothesis *κ* = 0 is plotted against *n*. This hypothesis can be rejected with more than 95% confidence for data points beneath the dashed line.
(TIF)

Figure S7 Analogous to Figure 4, but for *N*_lim_ = 1,024 bacteria and *T*_exp_ = 40 generations. (A) Fractions of isolates are plotted versus the logarithm of the selection coefficient (as inferred from the branching process) for four low antibiotic concentrations. Vertical dashed lines show the boundaries between the logarithmic bins used in the analysis. The distribution shifts towards smaller values as the antibiotic concentration increases, while a group of very fit mutants persists near the maximum selection coefficient *s* = 1. (B) Likelihood analysis of the estimated shape parameter (*κ*_e_) for the distribution of selection coefficients. The ML estimate *κ*_e_ is plotted against the number of beneficial mutants (ranked by their fitness effect) that were included for estimation (*n*) by using a fitness threshold (*w*_c_). Because each value of *n* corresponds to a range of values for *w*_c_, there is also a range of *κ*_e_ associated with every *n*. (C) The *P*-value corresponding to the hypothesis *κ* = 0 is plotted against *n*. This hypothesis can be rejected with more than 95% confidence for data points beneath the dashed line.
(TIF)

Figure S8The probability of parallel evolution (*P*_2_) and the fixation probability of the fittest mutant (*P*_max_) as inferred from the selection coefficients, are plotted versus antibiotic concentration. We also plot the prediction for the probability of parallel evolution in the case of a Gumbel class distribution, 
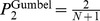
, where *N* is the number of beneficial mutants [20]. Note that, unlike the Gumbel case, we predict an increase in the frequency of parallel evolution events with increasing antibiotic concentration.(TIF)

Table S1 Mutation spectrum of the Mutazyme system. Mutation spectrum of the Mutazyme system used for PCR mutagenesis in our experiments. Sequenced isolates were recovered from an unselected library (0.00 µg Ctx/mL).
(DOCX)

Table S2 Identified unique mutations in TEM-1 β-lactamase ranked by their Ctx resistance. The table lists whether the adaptive mutations have previously been identified in clinical isolates (according to http://www.lahey.org/studies/temtable.asp; version 17 November 2011) or in laboratory experiments (according to [25]). The table also indicates how often a mutation has been sampled and in which MIC category it has been identified. Note that the listed frequencies refer to MIC measurements of different independent isolates and a particular genotype can consequently be identified in multiple MIC categories. MICs were determined directly after isolation, while IC99.99 levels were established after transformation into an isogenic background. Improvement is calculated by dividing the IC99.99 of the mutants by the IC99.99 of pACTEM1.
(DOCX)

Table S3 Probability of parallel evolution (*P*_2_) and fixation of largest-effect mutation (*P*_max_). Estimates are obtained from selection coefficients at various antibiotic concentrations.
(DOCX)

## References

[1] Toprak E, Veres A, Michel J-B, Chait R, Hartl DL (2012). Evolutionary paths to antibiotic resistance under dynamically sustained drug selection.. Nat Genet.

[2] Salverda MLM, Dellus E, Gorter FA, Debets AJM, Van der Oost J (2011). Initial mutations direct alternative pathways of protein evolution.. PLoS Genet.

[3] Sniegowski PD, Gerrish PJ (2010). Beneficial mutations and the dynamics of adaptation in asexual populations.. Phil Trans R Soc B.

[4] Weinreich DM, Delaney NF, DePristo MA, Hartl DL (2006). Darwinian evolution can follow only very few mutational paths to fitter proteins.. Science.

[5] Orr HA (1998). Testing natural selection versus genetic drift in phenotypic evolution using quantitative trait locus data.. Genetics.

[6] Orr HA (2003). The distribution of fitness effects among beneficial mutations.. Genetics.

[7] Orr HA (2010). The population genetics of beneficial mutations.. Phil Trans R Soc B.

[8] Gillespie JH (1984). Molecular evolution over the mutational landscape.. Evolution.

[9] Kassen R, Bataillon T (2006). Distribution of fitness effects among beneficial mutations prior to selection in experimental populations of bacteria.. Nat Genet.

[10] MacLean RC, Buckling A (2009). The distribution of fitness effects of beneficial mutations in *Pseudomonas aeruginosa*.. PLoS Genet.

[11] Rokyta DR, Joyce P, Caudle SB, Wichman HA (2005). An empirical test of the mutational landscape model of adaptation using a single-stranded DNA virus.. Nat Genet.

[12] Sanjuán R, Moya A, Elena SF (2004). The distribution of fitness effects caused by single-nucleotide substitutions in an RNA virus.. Proc Natl Acad Sci U S A.

[13] McDonald MJ, Cooper TF, Beaumont HJE, Rainey PB (2010). The distribution of fitness effects of new beneficial mutations in *Pseudomonas fluorescence*.. Biol Lett.

[14] Joyce P, Rokyta DR, Beisel CJ, Orr HA (2008). A General Extreme Value Theory Model for the Adaptation of DNA Sequences Under Strong Selection and Weak Mutation.. Genetics.

[15] Bataillon T, Zhang T, Kassen R (2011). Cost of Adaptation and Fitness Effects of Beneficial Mutations in *Pseudomonas fluorescens*.. Genetics.

[16] Rokyta DR, Beisel CJ, Joyce P, Ferris MT, Burch CL (2008). Beneficial Fitness Effects Are Not Exponential for Two Viruses.. J Mol Evol.

[17] Jain K, Seetharaman S (2011). Multiple Adaptive Substitutions During Evolution in Novel Environments.. Genetics.

[18] Neidhart J, Krug J (2011). Adaptive Walks and Extreme Value Theory.. Phys Rev Lett.

[19] Jain K (2011). Number of adaptive steps to a local fitness peak.. EPL.

[20] Orr HA (2005). The probability of parallel evolution.. Evolution.

[21] Orr HA (2003). A minimum on the mean number of steps taken in adaptive walks.. J Theor Biol.

[22] Fogle CA, Nagle JL, Desai MM (2008). Clonal Interference, Multiple Mutations and Adaptation in Large Asexual Populations.. Genetics.

[23] Park S-C, Simon D, Krug J (2010). The speed of evolution in large asexual populations.. J Stat Phys.

[24] Perfeito L, Fernandes L, Mota C, Gordo I (2007). Adaptive mutations in bacteria: high rate and small effects.. Science.

[25] Salverda MLM, de Visser JAGM, Barlow M (2010). Natural evolution of TEM-1 beta-lactamase: experimental reconstruction and clinical relevance.. FEMS Microbiol Rev.

[26] Barlow M, Hall BG (2002). Predicting evolutionary potential: *In vitro* evolution accurately reproduces natural evolution of the TEM beta-lactamase.. Genetics.

[27] Stemmer WPC (1994). Rapid evolution of a protein *in vitro* by DNA shuffling.. Nature.

[28] Davison AC, Smith RL (1990). Models for exeedances over high thresholds.. J R Stat Soc B (Methodological).

[29] Stevens KE, Sebert ME (2011). Frequent Beneficial Mutations during Single-Colony Serial Transfer of *Streptococcus pneumoniae*.. PLoS Genet.

[30] Lalic J, Cuevas JM, Elena SF (2011). Effect of Host Species on the Distribution of Mutational Fitness Effects for an RNA Virus.. PLoS Genet.

[31] Romero PA, Arnold FH (2009). Exploring protein fitness landscapes by directed evolution.. Nat Rev Mol Cell Biol.

[32] Hall AR, Griffiths VF, MacLean RC, Colegrave N (2010). Mutational neighbourhood and mutation supply rate constrain adaptation in *Pseudomonas aeruginosa*.. Proc R Soc B.

[33] DePristo MA, Weinreich DM, Hartl DL (2005). Missense meanderings in sequence space: a biophysical view of protein evolution.. Nat Rev Genet.

[34] Huang W, Petrosino J, Hirsch M, Shenkin PS, Palzkill T (1996). Amino acid sequence determinants of beta-lactamase structure and activity.. J Mol Biol.

[35] Soskine M, Tawfik DS (2010). Mutational effects and the evolution of new protein functions.. Nat Rev Genet.

[36] Kryazhimskiy S, Rice DP, Desai MM (2012). Population Subdivision and Adaptation in Asexual Populations of *Saccharomyces cerevisiae*.. Evolution.

[37] Wang X, Minasov G, Shoichet BK (2002). Evolution of an antibiotic resistance enzyme constrained by stability and activity trade-offs.. J Mol Biol.

[38] Goldsmith M, Tawfik DS (2009). Potential role of phenotypic mutations in the evolution of protein expression and stability.. Proc Natl Acad Sci U S A.

[39] Deana A, Ehrlich R, Reiss C (1996). Synonymous codon selection controls in vivo turnover and mount of mRNA in *Escherichia coli* bla and ompA genes.. J Bacteriol.

[40] Zalucki YM, Gittins KL, Jennings MP (2008). Secretory sequence signal non-optimal codons are required for expression and export of beta-lactamase.. Biochem Biophys Res Comm.

[41] Duan JB, Wainwright MS, Comeron JM, Saitou N, Sanders AR (2003). Synonymous mutations in the human dopamine receptor D2 (DRD2) affect mRNA stability and synthesis of the receptor.. Hum Mol Genet.

[42] Lind PA, Berg OG, Andersson DI (2010). Mutational Robustness of Ribosomal Protein Genes.. Science.

[43] Vakulenko SB, Taibi-Tronche P, Toth M, Massova I, Lerner SA (1999). Effects on substrate profile by mutational substitutions at positions 164 and 179 of the class A TEMpUC19 beta-lactamase from *Escherichia coli*.. J Biol Chem.

[44] Orencia MC, Yoon JS, Ness JE, Stemmer WPC, Stevens RC (2001). Predicting the emergence of antibiotic resistance by directed evolution and structural analysis.. Nat Struct Biol.

[45] Negri M-C, Lipsitch M, Blázquez J, Levin BR, Baquero F (2000). Concentration-dependent selection of small phenotypic differences in TEM beta-lactamase-mediated antibiotic resistance.. Antimicrob Agents Chemother.

[46] Paulander W, Pennhag A, Andersson DI, Maisnier-Patin S (2007). *Caenorhabditis elegans* as a model to determine fitness of antibiotic-resistant *Salmonella enterica* serovar typhimuriu.. Antimicrob Agents Chemother.

[47] Andersson DI, Hughes D (2010). Antibiotic resistance and its cost: is it possible to reverse resistance?. Nat Rev Microb.

[48] Medeiros AA (1997). Evolution and dissemniation of beta-lactamases accelerated by generations of beta-lactam antibiotics.. Clin Infect Dis.

[49] Derrida B, Fannes M, Maes C, Verbeure A (1994). Non-self averaging effects in sums of random variables, spin glasses, random maps and random walks.. On three levels: Micro-, meso- and macro-approaches in physics.

[50] Gifford DR, Schoustra SE, Kassen R (2011). The length of adaptive walks is insensitive to starting fitness in *Aspergillus nidulans*.. Evolution.

[51] Schoustra SE, Bataillon T, Gifford DR, Kassen R (2009). The Properties of Adaptive Walks in Evolving Populations of Fungus.. PLOS Biol.

[52] Sun F (1995). The polymerase chain reaction and branching processes.. J Comp Biol.

[53] Patrick WM, Firth AE, Blackburn JM (2003). User-friendly algorithms for estimating completeness and diversity in randomized protein-encoding libraries.. Protein Eng.

[54] Ambler RP, Coulson AFW, Frere JM, Ghuysen JM, Joris B (1991). A standard numbering scheme for the class A beta-lactamase.. Biochem J.

[55] DeLano WL (2002). The PyMOL Molecular Graphics System.

[56] Jaffe A, Chabbert YA, Semonin O (1982). Role of porin proteins OmpF and OmpC in the permeation of beta-lactams.. Antimicrob Agents Chemother.

[57] Finkelstein M, Tucker HG, Veeh JA (1998). Confidence intervals for the number of unseen types.. Stat Prob Lett.

[58] Beisel CJ, Rokyta DR, Wichman HA, Joyce P (2007). Testing the extreme value domain of attraction for distributions of beneficial fitness effects.. Genetics.

[59] Pickands J (1975). Statistical inference using extreme order statistics.. Ann Stat.

[60] Choulakian V, Stephens MA (2001). Goodness-of-fit tests for the generalized pareto distribution.. Technometrics.

[61] Lang M, Ouarda TBMJ, Bobée B (1999). Towards operational guidelines for over-threshold modeling.. J Hydrol.

[62] Haldane JBS (1927). The mathematical theory of natural and artificial selection.. Proc Cambridge Phil Soc.

