# Supplementary figures and images for: Quantifying the Adaptive Potential of an Antibiotic Resistance Enzyme
Source: PLoS Genet. 2012 Jun 28;8(6):e1002783. doi: 10.1371/journal.pgen.1002783 (PMC3386231; doi:10.1371/journal.pgen.1002783)

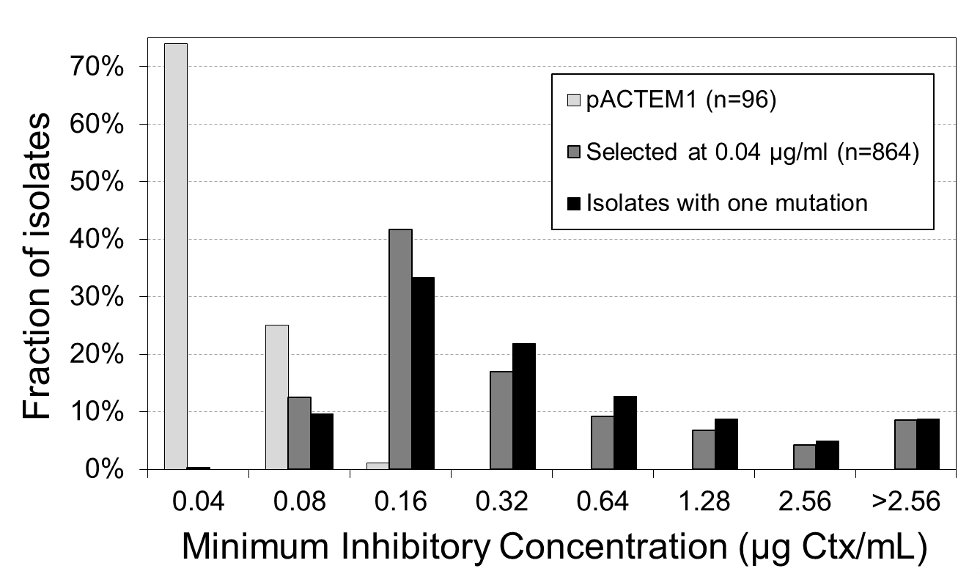

Supplement: Figure S1 — Histogram displaying the relative frequencies of MIC values of E. coli cells with plasmid-borne TEM β-lactamase that carry (1) the wild-type TEM-1 allele, (2) alleles from the mutated libraries that survived exposure to 0.04 µg Ctx/mL on plates (n = 864), and (3) alleles from the mutated libraries that carry only a single mutation (based on sequencing a sample of 310 isolates). MIC values were determined without transformation into a fresh isogenic background, and the potential presence of mutations outside the TEM gene was not eliminated in this assay. Note that Ctx concentrations are plotted on a log2 scale. (TIF) [file pgen.1002783.s001.tif]

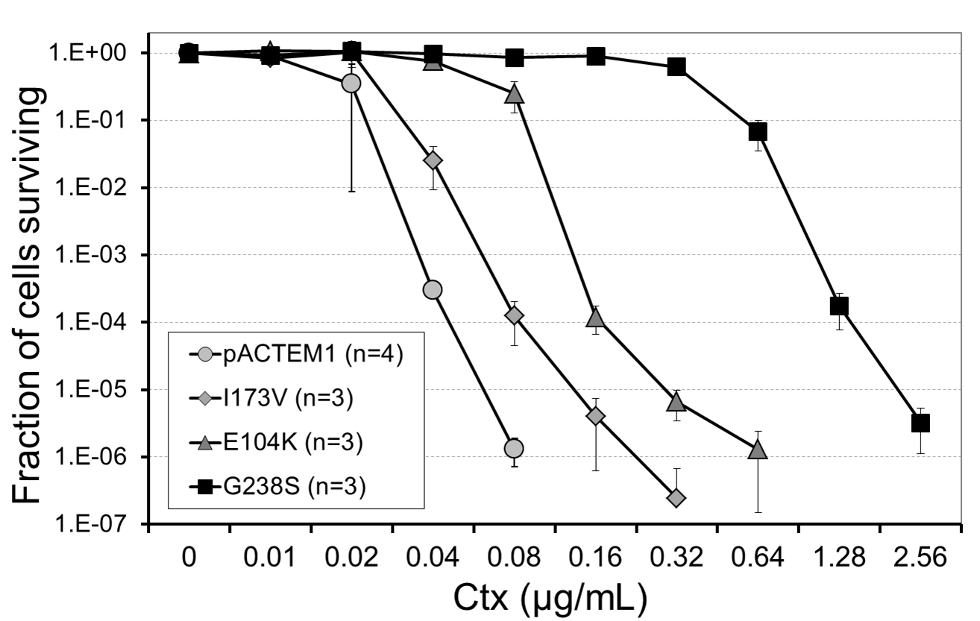

Supplement: Figure S2 — Survival of E. coli cells with plasmid-borne TEM β-lactamase at various Ctx concentrations. Survival is plotted as the fraction of surviving cells (± S.D.) on a logarithmic scale, and Ctx concentrations are plotted on a log2 scale. Comparison between cells carrying pACTEM1 and three mutants with a large (G238S), intermediate (E104K) and small-effect beneficial mutation (I173V), which shows the range of resistance effects encountered in this study. (TIF) [file pgen.1002783.s002.tif]

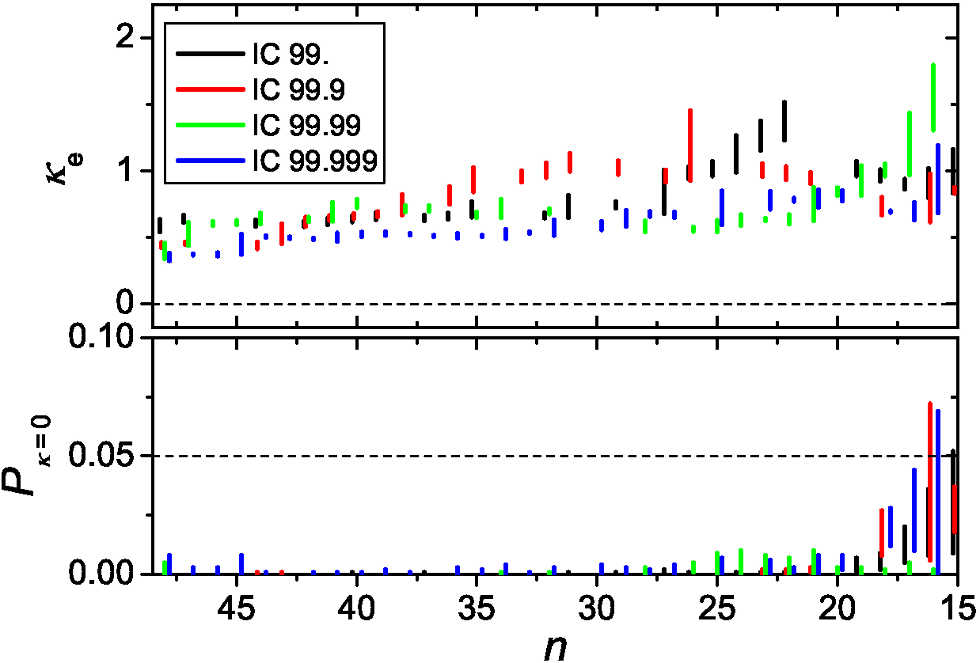

Supplement: Figure S3 — Likelihood analysis of the estimated shape parameter (κ e) of the GPD. In the upper panel, κ e is plotted against the number of beneficial mutants, ranked by their effect on resistance, that were used for the estimation (n) by using a threshold (w c). The IC99.99 was calculated as the Ctx concentration at which a fraction of 10−4 cells survived. This figure displays κ e corresponding to fractions of 10−2, 10−3, and 10−5 surviving cells to calculate the inhibitory Ctx concentration. Note that every n corresponds to a range of values of w c and thus to a range of estimates for κ e. The dashed line corresponds to κ = 0. κ e exceeds 0 for 15≤n≤50. The P-value corresponding to the hypothesis κ = 0 is plotted against n in the lower panel. The dashed line corresponds to the 95% confidence level. The hypothesis can be rejected with more than 95% confidence for all choices of wc and 15≤n≤48. (TIF) [file pgen.1002783.s003.tif]

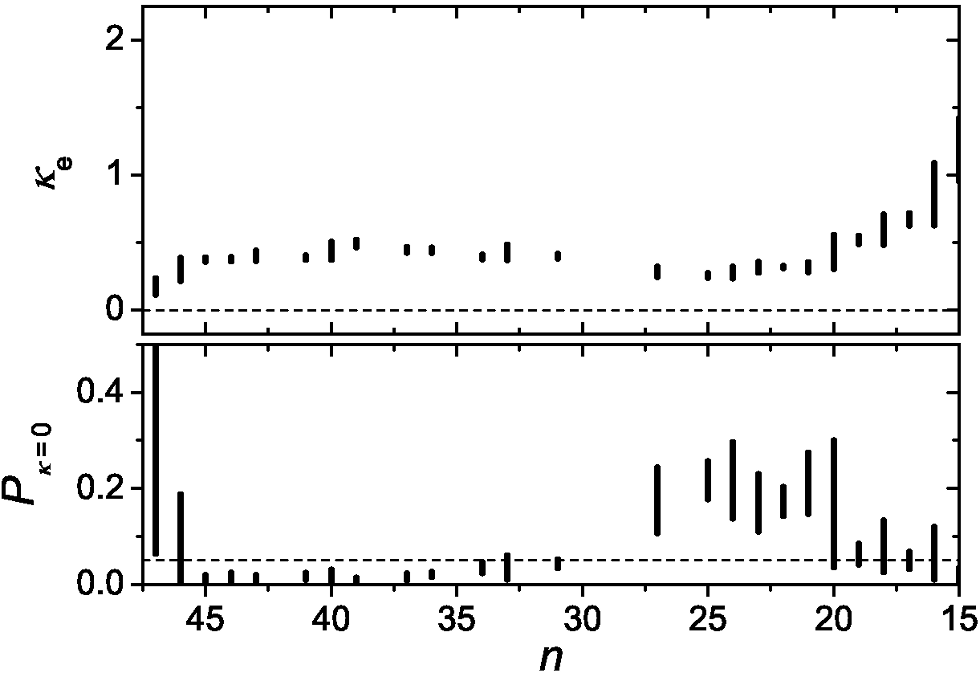

Supplement: Figure S4 — Analogous to Figure S3. The results of the likelihood analysis without the fittest mutant (G238S) are shown. This mutant is excluded to determine whether the condition κ>0 does not solely depend on this mutation. Compared to Figure S3, κ e shifts towards smaller values, but remains positive. The P-values obtained for the hypothesis κ = 0 are generally larger compared to the analysis with G238S, but remain below 0.05 (shown by the dashed line) for 30≤n≤45. (TIF) [file pgen.1002783.s004.tif]

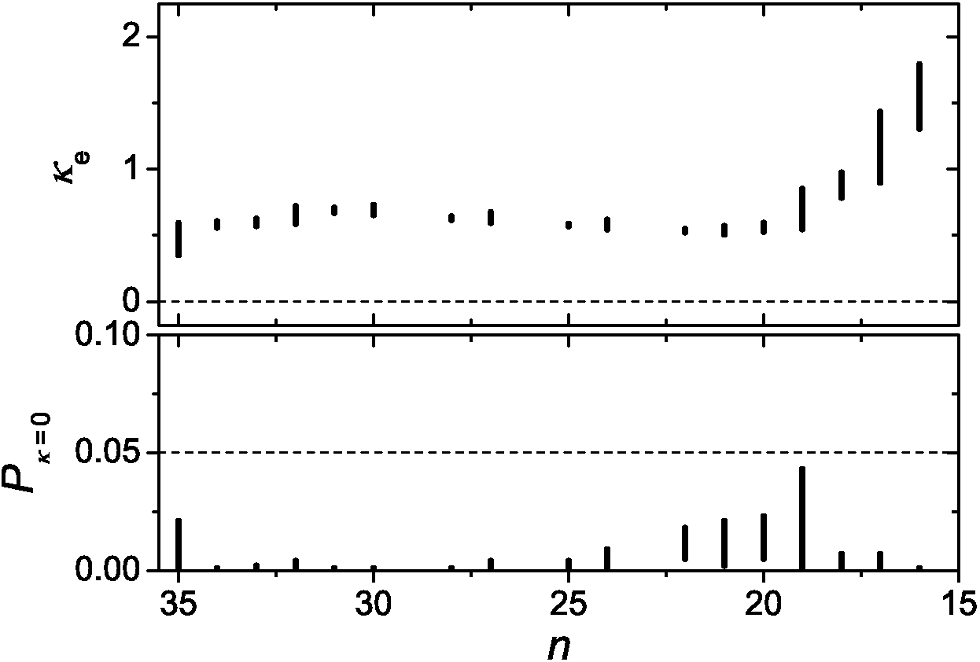

Supplement: Figure S5 — Analogous to Figure S3. The results of the likelihood analysis for only the non-synonymous mutations in the mature protein (n = 35) are shown. The removal of synonymous mutations does not affect the shape of the distribution. (TIF) [file pgen.1002783.s005.tif]

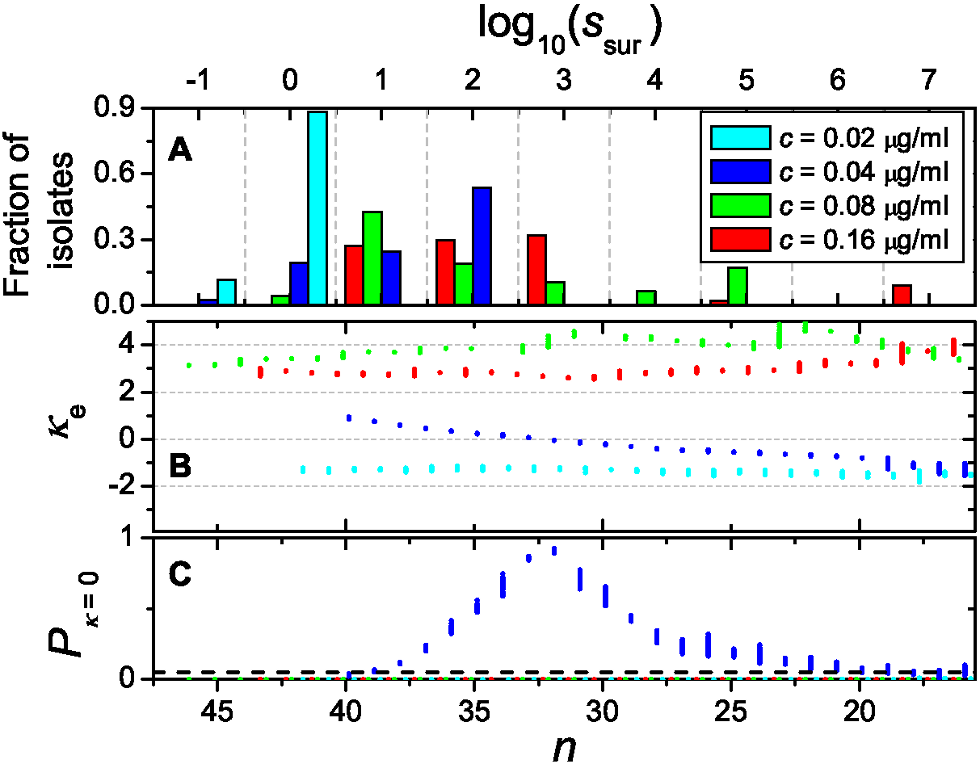

Supplement: Figure S6 — (A) Fractions of isolates are plotted versus the logarithm of the selection coefficient (as inferred from survival data) for four low Ctx concentrations. Vertical dashed lines show the boundaries between the logarithmic bins used in the analysis. (B) Likelihood analysis of the estimated shape parameter (κ e) for the distribution of selection coefficients. The ML estimate κ e is plotted against the number of ranked beneficial mutants that were included for estimation (n) by using a fitness threshold (w c). Because each value of n corresponds to a range of values for w c, there is also a range of κ e associated with every n. (C) The P-value corresponding to the hypothesis κ = 0 is plotted against n. This hypothesis can be rejected with more than 95% confidence for data points beneath the dashed line. (TIF) [file pgen.1002783.s006.tif]

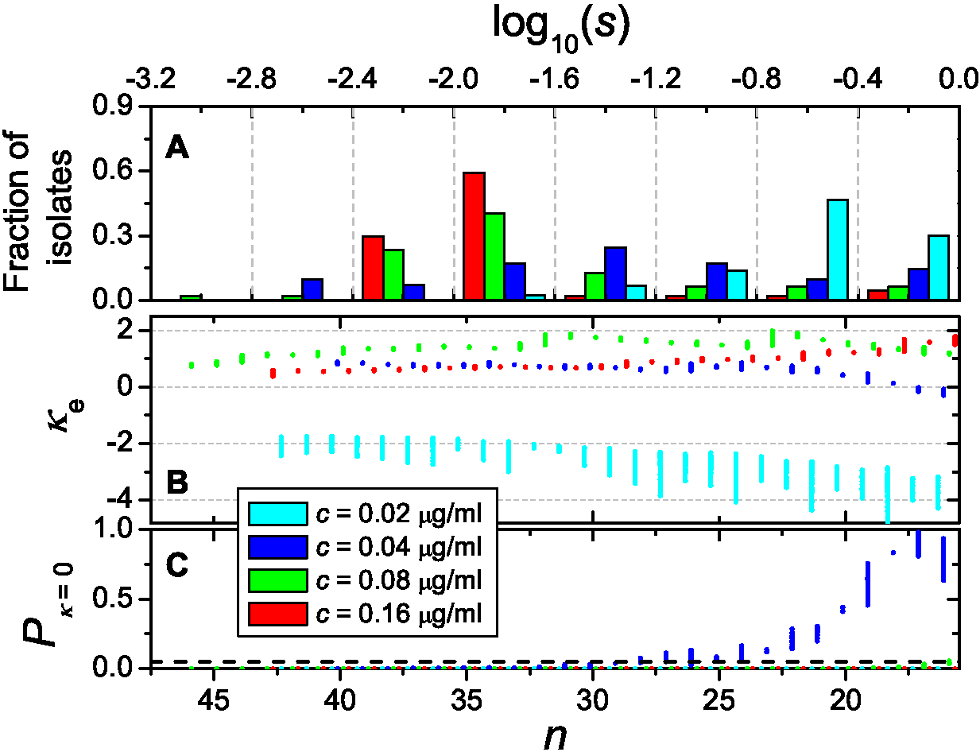

Supplement: Figure S7 — Analogous to Figure 4, but for N lim = 1,024 bacteria and T exp = 40 generations. (A) Fractions of isolates are plotted versus the logarithm of the selection coefficient (as inferred from the branching process) for four low antibiotic concentrations. Vertical dashed lines show the boundaries between the logarithmic bins used in the analysis. The distribution shifts towards smaller values as the antibiotic concentration increases, while a group of very fit mutants persists near the maximum selection coefficient s = 1. (B) Likelihood analysis of the estimated shape parameter (κ e) for the distribution of selection coefficients. The ML estimate κ e is plotted against the number of beneficial mutants (ranked by their fitness effect) that were included for estimation (n) by using a fitness threshold (w c). Because each value of n corresponds to a range of values for w c, there is also a range of κ e associated with every n. (C) The P-value corresponding to the hypothesis κ = 0 is plotted against n. This hypothesis can be rejected with more than 95% confidence for data points beneath the dashed line. (TIF) [file pgen.1002783.s007.tif]

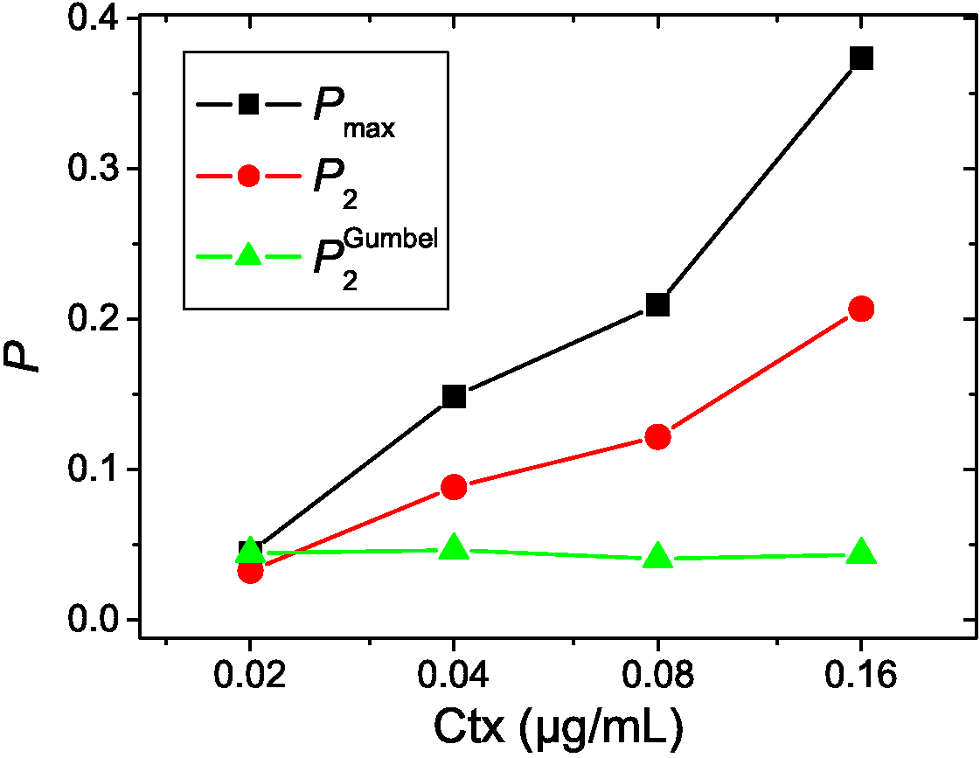

Supplement: Figure S8 — The probability of parallel evolution (P 2) and the fixation probability of the fittest mutant (P max) as inferred from the selection coefficients, are plotted versus antibiotic concentration. We also plot the prediction for the probability of parallel evolution in the case of a Gumbel class distribution, , where N is the number of beneficial mutants [20]. Note that, unlike the Gumbel case, we predict an increase in the frequency of parallel evolution events with increasing antibiotic concentration. (TIF) [file pgen.1002783.s008.tif]
