# Supplementary material for: Quantifying the Adaptive Potential of an Antibiotic Resistance Enzyme
Source: PLoS Genet. 2012 Jun 28;8(6):e1002783. doi: 10.1371/journal.pgen.1002783 (PMC3386231; doi:10.1371/journal.pgen.1002783)
Supplement: Table S1 — Mutation spectrum of the Mutazyme system. Mutation spectrum of the Mutazyme system used for PCR mutagenesis in our experiments. Sequenced isolates were recovered from an unselected library (0.00 µg Ctx/mL). (DOCX) [file pgen.1002783.s009.docx]

**Table S1**

|  | Number of mutations (%) |
| --- | --- |
| **Transitions** |  |
| A to G, T to C | 19 (17 %) |
| G to A, C to T | 41 (37 %) |
|  |  |
| **Transversions** |  |
| A to T, T to A | 28 (25 %) |
| G to C, C to G | 5 (5 %) |
| A to C, T to G | 7 (6 %) |
| G to T, C to A | 11 (10%) |
| Total | 111 |
