# Supplementary material for: Quantifying the Adaptive Potential of an Antibiotic Resistance Enzyme
Source: PLoS Genet. 2012 Jun 28;8(6):e1002783. doi: 10.1371/journal.pgen.1002783 (PMC3386231; doi:10.1371/journal.pgen.1002783)
Supplement: Table S2 — Identified unique mutations in TEM-1 β-lactamase ranked by their Ctx resistance. The table lists whether the adaptive mutations have previously been identified in clinical isolates (according to http://www.lahey.org/studies/temtable.asp; version 17 November 2011) or in laboratory experiments (according to [25]). The table also indicates how often a mutation has been sampled and in which MIC category it has been identified. Note that the listed frequencies refer to MIC measurements of different independent isolates and a particular genotype can consequently be identified in multiple MIC categories. MICs were determined directly after isolation, while IC99.99 levels were established after transformation into an isogenic background. Improvement is calculated by dividing the IC99.99 of the mutants by the IC99.99 of pACTEM1. (DOCX) [file pgen.1002783.s010.docx]

**Table S2**

| **Rank** | **Mutation** | **Replacement** | | **Clinical isolates ^*1^** | **Experimental evolution** | **Number of times identified**  **at MIC (μg Ctx/mL)** | | | | | | **Resistance level**  **IC99.99 (μg Ctx/mL)**  **± S.D.** | **Improvement** |
| --- | --- | --- | --- | --- | --- | --- | --- | --- | --- | --- | --- | --- | --- |
|  |  |  |  |  |  | **0.08** | **0.16** | **0.32** | **0.64** | **1.28** | **≥2.56** |  |  |
| 1 | *g706a* | G238S | | 34 | Yes |  |  |  |  |  | 36 | 1.41 ± 0.15 | 27 |
| 2 | *g707c* | G238A | | 0 | Yes |  |  |  |  | 2 |  | 0.566 ± 0.012 | 11 |
| 3 | *g713c* | R241P | | 0 | No |  |  |  |  | 3 |  | 0.363 ± 0.067 | 7.0 |
| 4 | *c484a* | R164S | | 30 | Yes |  |  |  |  | 3 |  | 0.320 ± 0.008 | 6.2 |
| 5 | *a710g* | E240G | | 0 | Yes |  |  |  | 20 | 26 |  | 0.247 ± 0.014 | 4.8 |
| 6 | *g304a* | E104K | | 46 | Yes |  |  | 2 | 4 |  |  | 0.171 ± 0.019 | 3.3 |
| 7 | *g707a* | G238D | | 1 | No |  |  | 9 | 4 |  |  | 0.160 ± 0.007 | 3.1 |
| 8 | *g485a* | R164H | | 19 | Yes |  |  | 4 | 5 | 2 |  | 0.151 ± 0.001 | 2.9 |
| 9 | *g703a* | A237T | | 9 | Yes |  |  | 3 | 1 |  |  | 0.149 ± 0.003 | 2.9 |
| 10 | *g508a* | A172T | | 0 | No |  |  | 5 |  |  |  | 0.147 ± 0.004 | 2.8 |
| 11 | *g640a* | V216I | | 0 | Yes | 1 |  |  |  |  |  | 0.138 ± 0.005 | 2.7 |
| 12 | *a506t* | E171V | | 0 | No |  | 5 | 1 |  |  |  | 0.136 ± 0.004 | 2.6 |
| 13 | *a790c* | S268R | | 0 | No |  |  |  | 1 |  |  | 0.134 ± 0.008 | 2.6 |
| 14 | *a710c* | E240A | | 0 | No |  |  | 2 |  |  |  | 0.134 ± 0.003 | 2.6 |
| 15 | *a305t* | E104V | | 0 | Yes |  |  |  | 1 |  |  | 0.131 ± 0.004 | 2.5 |
| 16 | *c509t* | A172V | | 0 | No | 5 | 7 |  |  |  |  | 0.131 ± 0.004 | 2.5 |
| 17 | *g706t* | G238C | | 0 | No |  |  | 1 |  |  |  | 0.128 ± 0.009 | 2.5 |
| 18 | *g255a* | G87* | | 0 | ^*2^ |  | 1 |  |  |  |  | 0.121 ± 0.016 | 2.3 |
| 19 | *g397a* | A135T | | 0 | No |  | 7 | 6 |  |  |  | 0.119 ± 0.005 | 2.3 |
| 20 | *g711t* | E240D | | 0 | Yes |  |  | 1 |  |  |  | 0.113 ± 0.008 | 2.2 |
| 21 | *t21a* | R9* | | 1 | ^*2^ |  | 2 |  |  |  |  | 0.101 ± 0.016 | 2.0 |
| 22 | *g713a* | R241H | | 0 | Yes |  |  | 1 |  |  |  | 0.097 ± 0.003 | 1.9 |
| 23 | *g261a* | E89* | | 2 | ^*2^ |  | 1 |  |  |  |  | 0.096 ± 0.016 | 1.9 |
| 24 | *t584a* | E197V | | 0 | No |  | 1 |  |  |  |  | 0.092 ± 0.003 | 1.8 |
| 25 | *t697a* | S235T | | 0 | Yes |  | 1 |  |  |  |  | 0.087 ± 0.010 | 1.7 |
| 26 | *a511g* | I173V | | 1 | Yes |  | 1 |  |  |  |  | 0.085 ± 0.011 | 1.6 |
| 27 | *g787c* | G267R | | 0 | Yes | 1 |  |  |  |  |  | 0.085 ± 0.004 | 1.6 |
| 28 | *c18t* | F8* | | 42 | ^*2^ | 2 | 1 |  |  |  |  | 0.081 ± 0.003 | 1.6 |
| 29 | *g22a* | V10I | | 0 | Yes | 1 |  |  |  |  |  | 0.081 ± 0.001 | 1.6 |
| 30 | *g45t* | A17* | | 0 | ^*2^ | 2 |  |  |  |  |  | 0.081 ± 0.002 | 1.6 |
| 31 | *c499a* | L169M | | 0 | No | 1 |  |  |  |  |  | 0.081 ± 0.003 | 1.6 |
| 32 | *c478a* | L162I | | 0 | No | 1 |  |  |  |  |  | 0.080 ± 0.006 | 1.6 |
| 33 | *a506g* | E171G | | 0 | No | 4 |  |  |  |  |  | 0.080 ± 0.002 | 1.5 |
| 34 | *g673a* | A227T | | 0 | No |  | 1 |  |  |  |  | 0.077 ± 0.006 | 1.5 |
| 35 | *g643a* | A217T | | 0 | No |  | 3 |  |  |  |  | 0.076 ± 0.001 | 1.5 |
| 36 | *g703t* | A237S | | 0 | Yes | 1 |  |  |  |  |  | 0.076 ± 0.003 | 1.5 |
| 37 | *t60c* | P22* | | 0 | ^*2^ | 1 |  |  |  |  |  | 0.075 ± 0.006 | 1.4 |
| 38 | *g411a* | L139* | | 0 | ^*2^ | 1 |  |  |  |  |  | 0.074 ± 0.004 | 1.4 |
| 39 | *a305c* | E104A | | 0 | Yes |  | 2 |  |  |  |  | 0.074 ± 0.003 | 1.4 |
| 40 | *a790g* | S268G | | 2 | Yes | 3 |  |  |  |  |  | 0.073 ± 0.003 | 1.4 |
| 41 | *g20a* | R9H | | 0 | No | 1 |  |  |  |  |  | 0.070 ± 0.001 | 1.4 |
| 42 | *c180t* | P62* | | 0 | ^*2^ | 1 |  |  |  |  |  | 0.069 ± 0.004 | 1.3 |
| 43 | *t208g* | F72V | | 0 | No |  | 1 |  |  |  |  | 0.069 ± 0.003 | 1.3 |
| 44 | *c712t* | R241C | | 0 | No | 1 |  |  |  |  |  | 0.065 ± 0.005 | 1.2 |
| 45 | *c526a* | R178S | | 0 | No |  |  | 1 |  |  |  | 0.063 ± 0.004 | 1.2 |
| 46 | *t129c* | C45* | | 0 | ^*2^ | 1 |  |  |  |  |  | 0.062 ± 0.008 | 1.2 |
| 47 | *a11g* | Q6R | | 0 | Yes | 1 |  |  |  |  |  | 0.059 ± 0.002 | 1.1 |
| 48 | *a645t* | A217* | | 0 | ^*2^ |  | 1 |  |  |  |  | 0.055 ± 0.003 | 1.1 |
| **Total number of isolates with one substitution** | | | | | | **31** | **35** | **36** | **36** | **36** | **36** |  |  |
|  | | | | | |  |  |  |  |  |  |  |  |
| - | none | pACTEM1 | | - | - | 23 | 29 | 2 |  | 1 |  | 0.052 ± 0.002 | 1.0 |
| - | Multiple mutations | | | - | - | 3 | 7 | 6 | 5 | 6 | 16 | - | - |
| ^*3^ | *c54t* | | C20* | 0 | ^*2^ | 1 |  |  |  |  |  | 0.049 ± 0.006 | 0.9 |
| ^*3^ | *g606a* | | R204* | 0 | ^*2^ | 1 |  |  |  |  |  | 0.047 ± 0.006 | 0.9 |
| ^*3^ | *g628a* | | E212K | 0 | No | 1 |  |  |  |  |  | 0.046 ± 0.006 | 0.9 |
| ^*3^ | *c249t* | | D85* | 0 | ^*2^ |  | 1 |  |  |  |  | 0.040 ± 0.008 | 0.8 |
| *-* | none | pACSE3 | | - | - |  |  |  |  |  |  | 0.033 ± 0.003 | 0.6 |
| **Total number of sequenced isolates** | | | | | | **58** | **72** | **44** | **41** | **43** | **52** |  |  |

*1. Number of lines present in 179 characterized alleles.

*2. The record on silent mutations is incomplete.

*3. These mutants were not beneficial according to the IC99.99 assay.
