# Supplementary material for: Quantifying the Adaptive Potential of an Antibiotic Resistance Enzyme
Source: PLoS Genet. 2012 Jun 28;8(6):e1002783. doi: 10.1371/journal.pgen.1002783 (PMC3386231; doi:10.1371/journal.pgen.1002783)
Supplement: Table S3 — Probability of parallel evolution (P 2) and fixation of largest-effect mutation (P max). Estimates are obtained from selection coefficients at various antibiotic concentrations. (DOCX) [file pgen.1002783.s011.docx]

**Table S3**

| **µg Ctx/mL** | **0.02** | **0.04** | **0.08** | **0.16** |
| --- | --- | --- | --- | --- |
| ***P_2_*** | 0.033 | 0.088 | 0.122 | 0.207 |
| ***P_max_*** | 0.045 | 0.149 | 0.209 | 0.374 |
